# Antagonistic Strain *Bacillus velezensis* JZ Mediates the Biocontrol of *Bacillus altitudinis* m-1, a Cause of Leaf Spot Disease in Strawberry

**DOI:** 10.3390/ijms25168872

**Published:** 2024-08-15

**Authors:** Li Zhang, Zirui Liu, Yilei Pu, Boyuan Zhang, Boshen Wang, Linman Xing, Yuting Li, Yingjun Zhang, Rong Gu, Feng Jia, Chengwei Li, Na Liu

**Affiliations:** 1 National Engineering Research Center of Wheat and Corn Further Processing, Henan University of Technology, Zhengzhou 450001, China; zhanglibio@haut.edu.cn; 2 College of Biological Engineering, Henan University of Technology, Zhengzhou 450001, China; 18637190851@163.com (Z.L.); puyi-lei1834@163.com (Y.P.); zhang_by2021@163.com (B.Z.); 17657315800@163.com (B.W.); lyt18692@163.com (Y.L.); zyj34800@163.com (Y.Z.); G19513317425@163.com (R.G.); mrjiafeng@163.com (F.J.); 3 School of International Education, Henan University of Technology, Zhengzhou 450001, China; xinglllllllm@163.com

**Keywords:** *Bacillus velezensis*, biocontrol, *Bacillus altitudinis*, Ca^2+^-ATPase, reactive oxygen species (ROS), strawberry

## Abstract

Biofertilizers are environmentally friendly compounds that can enhance plant growth and substitute for chemically synthesized products. In this research, a new strain of the bacterium *Bacillus velezensis*, designated JZ, was isolated from the roots of strawberry plants and exhibited potent antagonistic properties against *Bacillus altitudinis* m-1, a pathogen responsible for leaf spot disease in strawberry. The fermentation broth of JZ exerted an inhibition rate of 47.43% against this pathogen. Using an optimized acid precipitation method, crude extracts of lipopeptides from the JZ fermentation broth were obtained. The crude extract of *B. velezensis* JZ fermentation broth did not significantly disrupt the cell permeability of *B. altitudinis* m-1, whereas it notably reduced the Ca^2+^-ATPase activity on the cell membrane and markedly elevated the intracellular reactive oxygen species (ROS) concentration. To identify the active compounds within the crude extract, QTOF-MS/MS was employed, revealing four antimicrobial compounds: fengycin, iturin, surfactin, and a polyene antibiotic known as bacillaene. The strain JZ also produced various plant-growth-promoting substances, such as protease, IAA, and siderophore, which assists plants to survive under pathogen infection. These findings suggest that the JZ strain holds significant potential as a biological control agent against *B. altitudinis*, providing a promising avenue for the management of plant bacterial disease.

## 1. Introduction

Chemical pesticides and field management are widely used in agriculture and forestry to control diseases. However, owing to a pathogen’s natural persistence and various environmental factors, complete prevention and control may not be achieved by field management [1]. Therefore, the need for sustainable and environmentally friendly practices to enhance agricultural productivity is a pressing priority.

Many researchers are seeking to address this problem by developing sustainable means to increase agricultural productivity. An effective sustainable approach to increase agricultural yield is the use of microorganisms instead of chemical fertilizers, which are termed biofertilizers [2]. Biofertilizers are considered to be greener than chemical or physical technologies but are less stable as they depend on microbial adaptability to the local environment. Recently, biofertilizers have received considerable attention because of the potential to achieve an improved survival rate for the inoculant microorganisms. Numerous applications for biofertilizers have been studied over a prolonged period, most notably to increase plant biomass and nutrient availability [3,4,5]. However, a growing body of research is focused on the use of biofertilizers in the biological management of plant diseases.

The majority of plant diseases are attributed to pathogenic fungi, and numerous inhibitory mechanisms have been elucidated, leading to the development of various agents for the control of fungal phytopathogens [6]. In contrast, the inhibitory mechanisms against bacterial diseases remain poorly understood, and effective control agents for bacterial diseases are scarce [7]. Particularly concerning is the absence of control agents for certain bacterial diseases affecting edible fungi [8]. The pursuit of biocontrol bacteria capable of inhibiting pathogenic bacteria is crucial for addressing this shortage and important for the general advancement and application of microbial resources [6,7,8].

*Bacillus velezensis* has been recognized for its ability to inhibit a broad spectrum of pathogens, including *Botrytis cinerea* (the etiological agent for gray mold), *Streptomyces* sp. (black star disease), and *Alternaria solani* (early blight) [9,10]. Recent studies have demonstrated that *B. velezensis* not only possesses broad-spectrum antibacterial capabilities but also harbors genes that promote plant growth and augment plant immune activity [11]. Investigations into the secondary metabolites of *B. velezensis* have further validated its broad-spectral bacterial inhibition and plant-growth-promoting effects [12]. The antagonistic substances vary depending on the target pathogen [13,14].

*Bacillus altitudinis*, a Gram-positive bacterium, exhibits antifungal properties similar to *B. velezensis* and has been found to enhance plant root growth [11]. Nonetheless, in recent years, it has been identified as a causative agent of plant diseases, such as pomegranate seed rot in China, Thailand, and Morocco [15], blackleg and soft rot in apple and potato [12,16], and leaf burn in pear [15,17,18,19,20].

With regard to plant disease prevention and control, while certain *Bacillus* species, such as *Bacillus subtilis* and *Bacillus amyloliquefaciens*, have well-established antagonistic activities against pathogenic fungi [21,22], there is a paucity of reports on the inhibitory activity and mechanisms of *Bacillus* against pathogenic bacteria. Despite reports of the pathogenicity of *B. altitudinis* in several countries, no strategies for its control have been documented to date. This study aimed to evaluate the inhibitory activity and mechanism of *B. velezensis* against the pathogenic bacterium *B. altitudinis*, with the objective of identifying a potential biocontrol agent for the management of plant diseases caused by *B. altitudinis* and gaining an improved understanding of the inhibitory mechanisms of *Bacillus* against pathogenic bacteria [15,18].

The strain *B. velezensis* JZ was isolated from healthy strawberry plants in our laboratory and is preserved in the Agricultural Microbial Strain Conservation Centre of China (ACCC) under the strain number ACCC 62529. *Bacillus altitudinis* m-1 was isolated from strawberry leaves infected with leaf spot disease. Both JZ and m-1 were isolated and subsequently identified by 16S rRNA gene sequencing. The antagonistic effect of JZ on m-1 was assessed using the Kirby–Bauer disc diffusion method (K-B test) [23]. A crude extract from the JZ fermentation broth was prepared through an acid precipitation method, optimizing the extraction process. This crude extract was then employed to antagonize m-1, and assays were conducted to detect alterations in cell permeability, intracellular reactive oxygen species (ROS) concentration, superoxide dismutase (SOD) activity, and Ca^2+^-ATPase activity. This work identifies a biological agent with the potential to effectively control bacterial disease and promote plant growth in strawberry.

## 2. Results

### 2.1. Isolation and Identification of B. velezensis JZ and B. altitudinis m-1

The screening process yielded 30 bacterial strains isolated from the roots of strawberry plants, among which, three demonstrated significant inhibitory effects against pathogenic bacteria. The fermentation broth of JZ exhibited the most potent antagonistic effect against *B. altitudinis*, with an inhibition rate of 47.43% (Table 1). In addition, the fermentation broth of JZ showed notable inhibitory activity against other common bacteria, including *Brevibacterium bifidum*, *Bacillus proteolyticus*, and *Pseudomonas bohemia*, with inhibition rates of 43.12%, 69.90%, and 35.99%, respectively. Given the superior inhibitory effect of JZ, this bacterium was deemed to have the potential for the biological control of pathogenic bacteria and thus, was selected for further identification and analysis.

**Figure 1:**
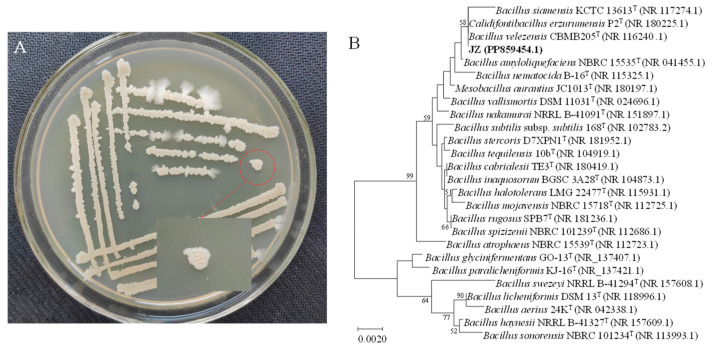
Colony morphology and genetic similarity of strain JZ. (**A**) Growth of JZ colony in PDA medium. (**B**) Dendrogram based on 16S rRNA gene sequences constructed using the neighbor-joining method.

*Bacillus velezensis* JZ presented a light brown-yellow hue and an opaque appearance when cultured on potato dextrose agar (PDA) medium at 37 °C. Initially, the colony surface was smooth with neat edges, but as incubation progressed, the surface became wrinkled with slightly uneven edges, rising in the middle and spreading in a cloudy pattern at the margins (Figure 1A). PCR amplification of the 16S rRNA gene from strain JZ produced a 1437 bp fragment. The DNA sequence was submitted to the GenBank database (accession No. PP859454). The nucleotide sequence of the JZ 16S rRNA gene bore a high similarity (99.72% homology) to that of *B. velezensis* strain CBMB 205 (Figure 1B). Consequently, the JZ strain could be identified as belonging to *B. velezensis*.

The results of physiological and biochemical tests indicated that strain JZ could catabolize maltose and lactose but not xylose and cellulose (Table 2). Strain JZ could hydrolyze starch and gelatin. The methyl red test and citrate utilization test were positive, whereas the H_2_S reaction was negative. Based on the cultural, morphological, and biochemical characteristics of strain JZ, it was tentatively identified as *Bacillus*.

**Figure 2:**
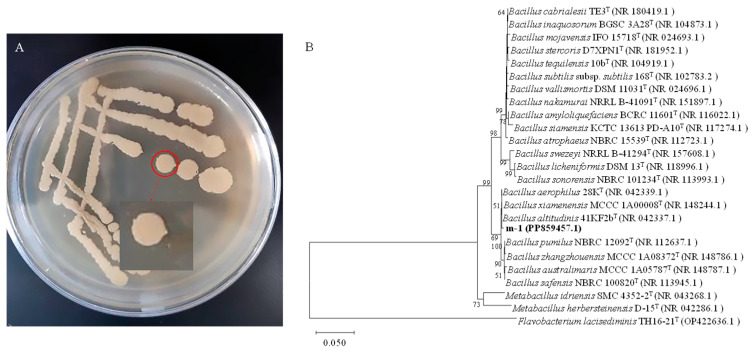
Colony morphology and genetic similarity of strain m-1. (**A**) Strain m-1 colonies grown on PDA medium. (**B**) Dendrogram based on 16S rRNA gene sequences constructed using the neighbor-joining method. *Flavobacterium lacisediminis* TH16-21^T^ (OP422636.1) was used as the outgroup.

The morphology of strain m-1 colonies was yellow, opaque, and moist when growing on PDA medium (Figure 2A). The surface of the colonies was smooth with neat edges. Gram staining showed that m-1 was a Gram-positive bacterium with a rod shape.

The 16S rRNA gene of strain m-1 was amplified using bacterial universal primers 27F and 1492R. The PCR amplification yielded a 1451 bp fragment. The DNA sequence was submitted to the GenBank database (accession No. PP859457). The nucleotide sequence of the m-1 16S rRNA gene showed high similarity (99.65% homology) to that of *B. altitudinis* strain 41KF2b (Figure 2B). Therefore, strain m-1 could be identified as belonging to *B. altitudinis*.

### 2.2. Assessment of the Inhibitory Activity of JZ Against m-1

**Figure 3:**
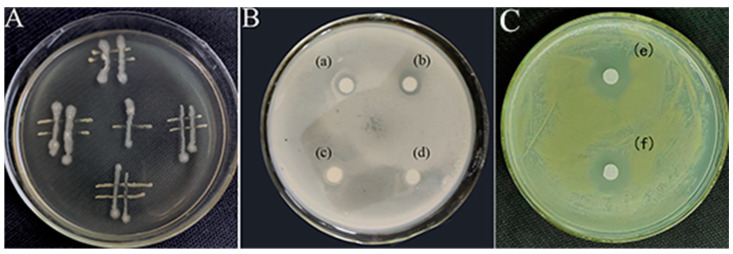
Interactions between the bacterial strains used in this study. (**A**) Streaking method. (**B**,**C**) K-B test: (a,b): a transparent ring is visible around the filter paper soaked with JZ fermentation broth; (c,d): no transparent ring is visible around the filter paper soaked with sterile water; (e,f): transparent ring is conspicuous around filter paper soaked with the extract from JZ fermentation broth.

The plate culture tests conducted in this study demonstrated an antagonistic interaction between the JZ and m-1 strains, with JZ exerting inhibitory effects on m-1 (Figure 3A). Clear zones of inhibition were observed around filter paper sheets saturated with JZ fermentation broth, whereas clear zones were absent around sterile filter paper sheets soaked in sterile water (Figure 3B), indicating that JZ produced compounds capable of suppressing the growth of m-1.

Similarly, the crude extract obtained after a fermentation period of 72 h and acid precipitation at pH 2.0 had a pronounced inhibitory effect on m-1, as evidenced by the formation of clear zones on the m-1 plates (Figure 3C). This finding indicated that the extraction process successfully concentrated the active inhibitory compounds, resulting in a more potent antimicrobial effect compared with that of the original fermentation broth.

### 2.3. Determination of the Minimum Inhibitory Concentration (MIC) of Crude Extract and Optimization of the Extraction Method

**Figure 4:**
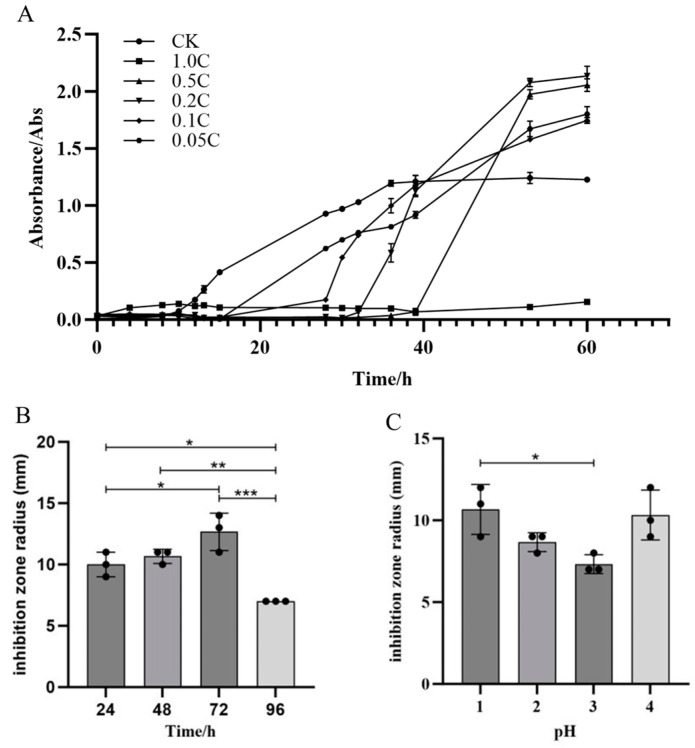
Growth curves of m-1, antibacterial activity, and optimization of the extraction. (**A**) Growth curves of m-1 cultures treated with different crude extract concentrations. The crude extract was diluted 1, 5, 10, and 20 times to generate the 0.5 C, 0.2 C, 0.1 C, and 0.05 C extracts, respectively; CK: no crude extract treatment. (**B**) Antibacterial activity of lipopeptide crude extracts. (**C**) Optimization of the pH for acid precipitation. * *p* < 0.05, ** *p* < 0.01, *** *p* < 0.001.

Crude extracts from JZ fermentation broth at concentrations of 0.05 C, 0.1 C, 0.2 C, 0.5 C, and 1.0 C were able to inhibit the growth of m-1, with varying degrees of inhibition observed among the concentrations (Figure 4A). The crude extract of JZ extended the growth lag phase of m-1 and inhibited growth for a certain period. In the control group, the lag phase of m-1 was approximately 8 h. In the experimental group, the lag phase was extended by approximately 8 h, 20 h, 24 h, and 30 h by the 0.05 C, 0.1 C, 0.2 C, and 0.5 C crude extracts, respectively. The m-1 culture inoculated with the 1.0 C crude extract did not enter the logarithmic growth phase after 60 h, indicating that the extract extended the lag phase by more than 50 h, potentially killing all m-1 colonies in the culture. Therefore, 0.05 C represented the MIC that inhibits m-1 growth in this experiment. During the experiment, the growth of m-1 was inhibited by several dilutions of the crude extract. However, after the inhibition period, m-1 growth resumed with a shortened logarithmic growth phase, suggesting a reduced cell generation time and uneven growth, particularly at higher concentrations of the inhibitory substances.

Optimization experiments showed that the crude extract with a fermentation time of 72 h (Figure 4B) exhibited the strongest bacterial inhibition. Acid precipitation optimization results indicated that the most potent inhibition occurred at pH 1.0 or 4.0, with an inhibition radius of 11 mm (Figure 4C). At pH 3.0, the inhibition radius was 7 mm, showing a weaker antibacterial effect compared with that for pH 1.0. These experimental phenomena demonstrate that different types and concentrations of antibacterial substances in different crude extracts resulted in differing antibacterial effects. The crude extract prepared with acid precipitation at pH 2.0 was selected for further experimentation.

### 2.4. Effect of Crude Extract on the Permeability and Ca^2+^-ATPase Activity of m-1 Cell Membranes

**Figure 5:**
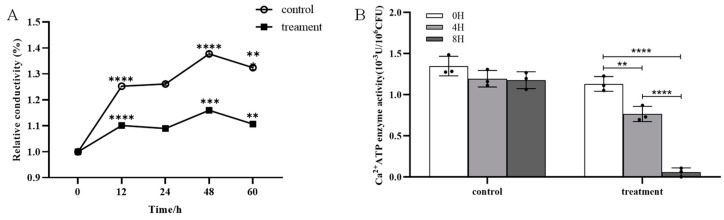
Conductivity and Ca^2+^-ATPase activity of m-1 treated with JZ crude extract. (**A**) Relative conductivity. Control: 9 mL PDB + 1 mL sterile water; treatment: 9 mL PDB + 1 mL 0.5 C crude extract. Significance represents the difference between the current and preceding time points. (**B**) Ca^2+^-ATPase activity. ** *p* < 0.01, *** *p* < 0.001, **** *p* < 0.0001.

No significant difference in the cell membrane permeability of m-1 was observed between the treatment group (exposed to JZ crude extracts) and the control group over a period of 60 h (Figure 5A). The conductivity fluctuations of the control and treatment were synchronous, showing the same increase and decrease. The results indicated that the crude extracts of JZ did not significantly alter the cell membrane permeability of m-1. 

The generation of a standard curve using the bacterial concentration and corresponding optical density (OD) values provides a quantitative method for the assessment of bacterial growth. The linear relationship given by the equation *y* = 3.8787 + 38.1814*x* with a high correlation coefficient of 0.9937 indicated a strong positive linear relationship between bacterial concentration and OD value. Thus, this curve was extremely reliable for the prediction of bacterial concentrations based on OD measurements.

The Ca^2+^-ATPase activity in the bacterial broth treated with crude extracts was significantly reduced compared with that of the control; after 4 h of treatment, the enzyme activity decreased by approximately 30% (Figure 5B). The enzyme activity approached zero at 8 h. This marked reduction in enzyme activity suggested a strong inhibitory effect of the crude extract on the m-1 Ca^2+^-ATPase activity in the cell membranes, which is critical for maintaining cellular calcium concentrations and overall cellular functions.

The foregoing findings suggested that, although the crude extract did not affect the cell membrane permeability of m-1, it significantly inhibited Ca^2+^-ATPase activity, potentially disrupting cellular processes that rely on calcium signaling. This inhibition might be a crucial mechanism by which the crude extract exerts its antibacterial effects against m-1.

### 2.5. Effect of Crude Extracts on Intracellular Rreactive Oxygen Species (ROS) and Superoxide Dismutase (SOD) Activity in m-1 Cells

**Figure 6:**
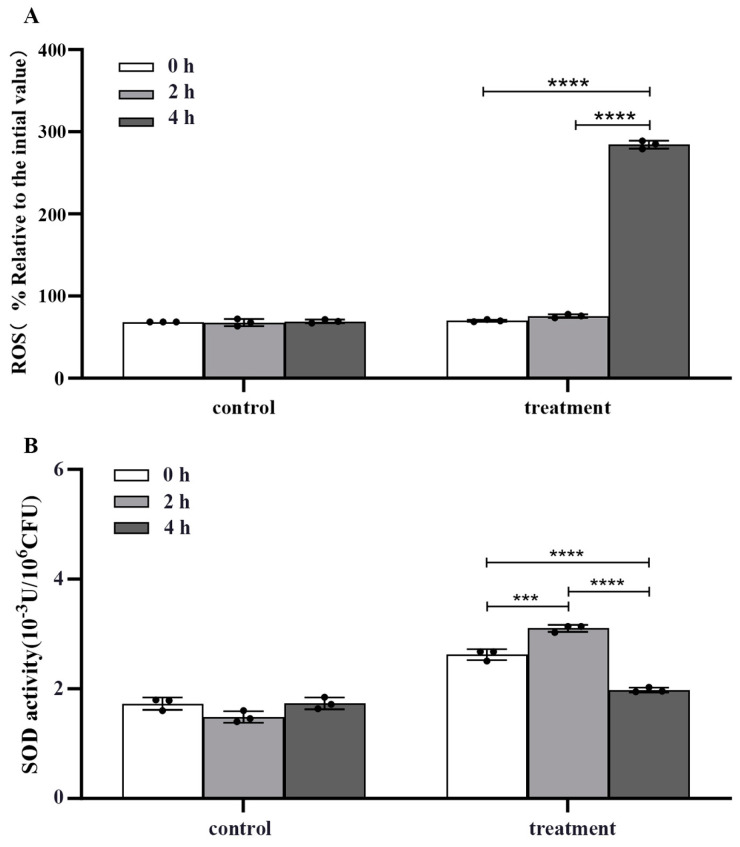
Reactive oxygen species (ROS) concentration and superoxide dismutase (SOD) activity of m-1 treated with JZ crude extract. (**A**) Intracellular ROS concentration. (**B**) SOD activity. *** *p* < 0.001, **** *p* < 0.0001.

The crude extract significantly elevated the ROS concentration in m-1 cells, as evidenced by a four-fold increase in fluorescence value after treatment for 4 h compared with that of the untreated control (*p* < 0.0001) (Figure 6A).

In conjunction with the increase in ROS concentration, a biphasic response in SOD activity following crude extract treatment was observed (Figure 6B). Initially, at 2 h after treatment, there was a significant increase in SOD activity, suggesting an attempt by the cells to mitigate the elevated ROS concentration by enhancing the dismutation of superoxide radicals to hydrogen peroxide and oxygen. However, at 4 h, the SOD activity decreased significantly compared with that of the control (*p* < 0.0001), indicating a potential depletion or inhibition of this critical antioxidant enzyme.

### 2.6. Semi-Preparative High-Performance Liquid Chromatography (HPLC) and Quadrupole Time-of-Flight/Tandem Mass Spectrometry (QTOF-MS/MS) of Substances with Antagonistic Activity in the JZ Crude Extract

**Figure 7:**
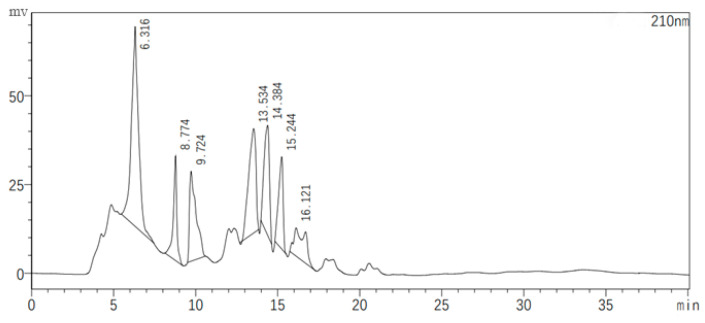
Semi-preparative HPLC chromatogram of the crude extract.

Figure 7 illustrates the chromatographic separation of seven classes of substances present in the JZ crude extract, with each peak representing a different sample collected at specific retention times. The antagonistic activity of these samples was evaluated, revealing that only the eluents corresponding to the second peak (8.774 min) and the fifth peak (14.384 min) exhibited inhibitory effects against bacterial growth. These active samples were then subjected to QTOF-MS/MS analysis for compound identification.

**Figure 8:**
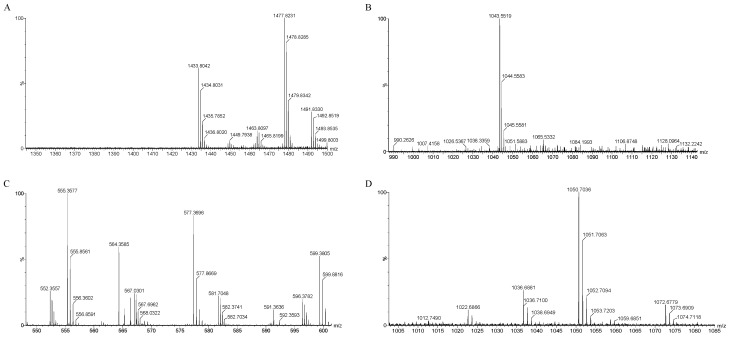
QTOF-MS/MS analysis of JZ crude extract. Spectra of (**A**) fengycin, (**B**) iturin, (**C**) bacillaene, (**D**) surfactin.

The mass spectrometry results, as depicted in Figure 8A–D, identified several compounds known for their antibacterial properties: fengycin, iturin, bacillaene, and surfactin (Table 3). Specifically, surfactin was detected with mass-to-charge ratio (*m*/*z*) values of 1022.68, 1036.68, and 1050.07/1051.07 (C14–C16). Iturin was identified at *m*/*z* 1043.55/1044.55. Fengycin was characterized by *m*/*z* values of 1449.79, 1463.80, 1477.82, and 1491.83 (C15–C17). Bacillaene, another notable compound, was identified at *m*/*z* 567.03 and 581.70.

## 3. Discussion

In this experiment, *B. velezensis* JZ isolated from the roots of healthy strawberry plants exhibited antagonistic properties against *B. altitudinis* m-1, which was isolated from leaf-blight-affected strawberry plants. During the antagonism assessment of *B. velezensis* JZ against *B. altitudinis* m-1, we observed that both the fermentation broth of JZ and the crude peptide extract derived from the fermentation broth exhibited inhibitory effects. Notably, the crude extract demonstrated a more pronounced inhibitory effect compared with that of the fermentation broth. In optimizing the extraction method for the crude extract, we determined that acid precipitation at different pH levels yielded crude extracts with significant inhibitory effects, although the coloration and precipitation state of these extracts differed. The crude extract significantly extended the growth lag phase of m-1 and following the inhibitory period induced by the crude extract, m-1 exhibited unbalanced growth upon recovery.

Previous studies have indicated that *Bacillus* species can modify the permeability of pathogenic fungal cells through mechanisms not only limited to cell membrane perforation but also including cell wall disruption [27]. However, the present experimental findings did not reveal any impact of the crude extract on the cell membrane permeability of *B. altitudinis* m-1. Research conducted by Banerjee et al., Arrebola et al., Lam et al., and Fan et al. [27,28,29,30] identified iturin and fengycin as the primary inhibitory substances produced by *Bacillus* spp. against pathogenic fungi. Genome sequencing of *B. velezensis* revealed the presence of genes associated with these two bacteriostatic compounds [30]. Using isotope tracing, Banerjee et al. observed that iturin and fengycin inhibit (1,3)-β-D-glucan synthase activity, thereby disrupting fungal cell wall synthesis [27]. Given that (1,3)-β-D-glucan synthase is a crucial enzyme in fungal cell wall synthesis, the antagonistic properties of iturin and fengycin are not specifically exerted on bacterial cells. In addition, beyond antimicrobial peptides, the genomes and metabolomes of *B. velezensis* and *B. subtilis* include chitinase and glucanase [31,32,33], enzymes capable of directly hydrolyzing fungal cell walls, an action not applicable to bacterial cell walls. The present analysis detected the presence of iturin and fengycin in the crude extract, elucidating why these compounds do not significantly damage bacterial cell membranes. This aligns with the experimental results showing no marked effect of the JZ crude extract on m-1 cell permeability, which explains the broad-spectral antifungal activity of antimicrobial peptides while exhibiting limited antibacterial efficacy against bacteria to some extent.

ATPases, located in cell and organelle membranes, play a pivotal role in bacterial energy metabolism, material transport, energy conversion, and information transfer. It is reported that the disruptive effect of 1-nonanol on *Aspergillus flavus* growth reflects decreases in succinate dehydrogenase, mitochondrial dehydrogenase, and ATPase activities, and the accumulation of ROS [34]. The present results indicated a significant reduction in Ca^2+^-ATPase activity on cell membranes of m-1 treated with the JZ crude extract, with the enzyme almost becoming inactivated after 8 h. This suggests that the crude extract may induce abnormal energy metabolism in m-1 cells. Huda et al. [35] studied a mutant strain overexpressing Ca^2+^-ATPase and observed elevated activities of three major antioxidant enzymes—chloramphenicol acetyltransferase, ascorbate peroxidase, and glutathione reductase—compared with those of the wild type under exposure to abiotic stresses. A decrease in Ca^2+^-ATPase activity could potentially affect the activity of intracellular antioxidant enzymes. In the present study, the JZ crude extract led to a decline in m-1 Ca^2+^-ATPase activity and an increase in m-1 intracellular ROS concentration. It is plausible that the reduced Ca^2+^-ATPase activity contributed to the increase in intracellular ROS concentration.

Following treatment with *Bacillus tequilensis* JK-11 culture filtrate, spore germination and mycelial dry weight of *Bipolaris sorokiniana* decreased, and the activities of antioxidant enzymes and crucial metabolic enzymes in mycelial cells were significantly impaired [36]. The present experimental results indicated a significant increase in SOD activity after crude extract treatment for 2 h, with the ROS concentration remaining comparable to that at 0 h. This suggested that, although the ROS concentration in m-1 cells increased initially at 2 h after crude extract treatment, sufficient SOD was produced by m-1 cells to counteract the increase, thereby maintaining a steady concentration at 2 h. However, a notable decrease in SOD activity was evident after treatment for 4 h, coinciding with a significant reduction in Ca^2+^-ATPase activity and a marked rise in ROS concentration, further substantiating the correlation between Ca^2+^-ATPase and antioxidant enzyme activities. The decrease in Ca^2+^-ATPase activity resulted in insufficient SOD production to eliminate ROS effectively, leading to a dramatic rise in ROS concentration.

In research on plant disease control, modulation of ROS concentrations represents a crucial strategy. For instance, *Bacillus* spp. can target pathogens, inducing ROS accumulation and subsequently triggering fungal apoptosis [37]. Xu et al. reported that changes in mitochondrial membrane potential could play a role in the induction of cell metabolism. The accumulation of excessive ROS leads to damage to intracellular DNA and efflux from cell membranes [38]. In the present study, the JZ crude extract inhibited m-1 growth, a process linked to the elevated intracellular ROS concentration; this surge in ROS concentration is a vital factor contributing to the suppression of pathogenic bacterial activity.

Zhou et al. reported that *Bacillus cereus* YN917 produced indole-3-acetic acid (IAA), 1-aminocyclopropane-1-carboxylate deaminase, siderophores, protease, amylase, cellulase, and β-1,3-glucanase, and harbored mineral phosphate decomposition activity [39]. Xu et al. showed that *B. subtilis* YB-04 could secrete protease, amylase, cellulase, siderophores, and IAA [40]. It is reported that different genera have different enzyme production capacities. In the present study, *B. velezensis* strain JZ produced extracellular enzyme activity and various plant-growth-promoting substances, such as protease, siderophore, and IAA, which assists plants to survive under pathogen infection.

In conclusion, we identified the strain *B. velezensis* JZ antagonistic to *B. altitudinis* m-1, a causative agent of plant diseases. Despite the congeneric relationship of the two species, both the crude extract and fermentation solution of JZ inhibited m-1 growth, resulting in decreased Ca^2+^-ATPase activity in the cell membranes of m-1, initial elevation followed by a decline in SOD activity and accumulation of intracellular ROS. We optimized the acid precipitation method for the peptide crude extracts, an advancement that will be valuable for future preparations of peptide crude extracts. This study investigated the possible mode of action of *B. velezensis* JZ against *B. altitudinis* m-1 and evaluated the effects of JZ crude extract on the pathogen control. It is concluded that the strain *B. velezensis* JZ could be used in a biocontrol management program for sustainable agriculture.

## 4. Materials and Methods

### 4.1. Isolation and Identification of B. velezensis JZ and B. altitudinis m-1

Plant samples for screening the isolated antagonistic bacteria were collected from healthy strawberry plants grown in Shangqiu, Henan Province. The pathogens involved in this study were all kept in the laboratory of the School of Biological Engineering, Henan University of Technology. The medium was potato dextrose agar (PDA) medium (2% glucose, 20% potato, 2% agar, pH 7.0) [41]. The strains were grown on PDA plates using the streak plate technique and incubated at 37 °C for 24 h. Single colonies were taken for Gram staining and the basic characteristics of the strains were observed under the microscope. According to Bergey’s Manual of Systematic Bacteriology, their physiological and biochemical characteristics were analyzed through citrate utilization test, gelatin liquefaction test, carbon source utilization test, H_2_S gas production test, methyl red test, and starch hydrolysis test. The 16S rRNA gene sequence of the extracted DNA was amplified through polymerase chain reaction (PCR) with two bacterial universal primers, namely 27F and 1492R. The PCR products were sequenced by Sangon Biotech (Shanghai, China). Mega software (version 7.0) was used for constructing a phylogenetic tree using the neighbor-joining method.

### 4.2. Identification of the Inhibitory Activity of JZ Cells against m-1 Cells

The K-B test determined the inhibitory effect of the fermentation broth of JZ on m-1. The purified JZ was inoculated into the PDB (2% glucose, 20% potato, pH 7.0) culture and incubated at 37 °C, 120 rpm for 72 h to obtain JZ fermentation broth. The sterile JZ fermentation broth was filtered through the 0.45 μm and 0.22 μm pore size filter membrane in a clean bench. The purified m-1 was picked into the PDB culture at 37 °C, 120 rpm for 24 h. Then, the m-1 samples were diluted with distilled water and an appropriate amount of diluted solution was spread on PDA medium. Filter paper discs (5 mm) soaked with JZ sterile fermentation solution with 20 μL were applied to the plates containing bacteria, and sterile water was used as the control. After 24 h of incubation at 37 °C, the inhibition effect was observed. Each treatment was repeated three times.

### 4.3. Determination of the Antibacterial Activity of Crude Extracts and Optimization of the Extraction Method

The crude extract of JZ fermentation broth was extracted according to the method of Luo [42], and the optimization of acid precipitation pH and fermentation time was carried out based on the single variable principle to maximize the bacterial inhibitory effect of the crude extract in this experiment.

#### 4.3.1. Optimization of pH in Acid Precipitation

The strain of JZ was inoculated in PDB and fermented in a shaker at 37 °C, 120 rpm for 72 h. The fermentation broth was centrifuged at 8000 rpm for 10 min and the supernatant was filtered through a 0.45 μm membrane to obtain the fermentation broth supernatant. After dividing the supernatant into four parts and using HCl to modify pH to 1.0, 2.0, 3.0, and 4.0, it stood at 4 °C for the entire night. The samples were then centrifuged at 9000 rpm for 10 min, the precipitate was collected and dissolved in 60% methanol (fermentation broth: dissolution solution = 100:1 v/v). The solution was adjusted to pH 7.0 and centrifuged at 12,000 rpm for 10 min, and the supernatant was collected as a crude extract. Then, the crude extract was filtered through a 0.22 μm membrane to obtain acid precipitations at four different pH values.

#### 4.3.2. Optimization of Fermentation Time and Activity Determination of Crude Extracts

The JZ strain was fermented for 24 h, 48 h, 72 h, and 96 h at 37 °C and 120 rpm in PDB medium. The pH of the supernatant was adjusted to 2.0 by HCl and the rest of the conditions remained unchanged, resulting in crude extracts with four different fermentation times. Filter paper discs were soaked in four crude extracts, each of 20 μL. The radius of the inhibition circle of the crude extracts was determined according to the K-B test of Section 4.2.

### 4.4. Minimum Inhibitory Concentration of Crude Extract on m-1 Cells

The MIC of the crude extracts was defined as the concentration that significantly prolonged the m-1 lag phase referring to Park et al. and Wang et al. [43,44]. The growth curves were determined using turbidimetry to determine the lag phase of growth of m-1 at different concentrations of the crude extract.

The m-1 was inoculated in PDB and incubated in a shaker at 37 °C and 120 rpm for 12 h as seed solution. The crude extract of JZ fermentation broth was diluted 1, 5, 10, and 20 times to 0.5 C, 0.2 C, 0.1 C, and 0.05 C (C represents the concentration of the stock solution from the extracted crude extract at a ratio of 1:100 v/v). The samples were divided into one control group (CK) and five treatment groups, as in Table 4, and then incubated in a shaker at 37 °C and 120 rpm for 0, 4, 8, 10, 12, 13.5, 15, 28, 30, 32, 36, 39, 53, and 60 h. Samples were measured for absorbance at λ = 600 nm using a UV spectrophotometer. The experiment was repeated three times.

### 4.5. Effect of Crude Extract on the Permeability of m-1 Cell Membranes

According to the method of Zhang et al. to determine cell membrane permeability, m-1 was inoculated in PDB at 37 °C and 120 rpm for 12 h until the logarithmic growth phase, and then, centrifuged at 6000 rpm to extract the precipitate [45]. The precipitate was washed three times with PBS buffer (pH 7.0). The bacteria were suspended with distilled water. Tenfold MIC crude extract was added to the bacterial suspension in a 10% volume ratio, with sterile water serving as the control group. The sample was cultivated in a shaker at 37 °C and 120 rpm. The conductivity of samples was determined every 12 h. The process was repeated three times.

### 4.6. Effect of Crude Extract on m-1 Cell Membrane Ca^2+^-ATPase Activity

The ATPase activity on cell membrane was determined using the ultra-micro Ca^2+^-ATPase test kit (Nanjing Jiancheng Bioengineering Institute, Nanjing, China). The method was appropriately modified according to the suggestion of the supplier. The unit of enzyme activity for this experiment was specified as one unit of enzyme activity per million bacteria per hour for Ca^2+^-ATPase decomposition of ATP to produce 1 μmol of inorganic phosphate.

Before enzyme activity assays, the bacterial concentration (OD_600_ value) was determined using UV spectrophotometry and counted via plate counting, establishing a standard curve between OD_600_ values and bacterial suspensions in milliliters per liter. Protein was extracted using ultrasonication. The cells were crushed in an ice bath to obtain the sample solution containing Ca^2+^-ATPase by ultrasonic crusher. The crushing conditions were set as follows: power 15%, working time 5 s, stopping time 5 s, and total time 10 min. The sample solution was assayed for enzyme activity according to the instructions of the test kit.

#### 4.6.1. Preparation of the Reaction System

Strain m-1 was inoculated into PDB medium at a 5% seed ratio, while 10% crude extract, which was 10 times the minimum inhibitory concentration (MIC), was added in a 10% proportion (v/v). A control group was set up using sterilized water. At 37 °C and 120 rpm for cultivation, samples were collected at 0, 4, and 8 h.

#### 4.6.2. Detection of Enzyme Activity

A 1 mL sample of bacterial solution of the reaction system was centrifuged at 6000 rpm for 5 min and then the supernatant was removed and the precipitate was collected. A total of 2 mL of Tris-HCl was added to resuspend the bacteria. The cells were then crushed in an ice bath using an ultrasonic crusher and centrifuged at 9000 rpm for 10 min at 4 °C. The activity of Ca^2+^-ATPase was measured with supernatant. Samples were taken three times in parallel with control and treatment.

The samples were treated with chemical reagents according to the test kit instructions. The OD value of the samples was measured using a UV spectrophotometer. The corresponding enzyme activity was then calculated according to the rules.

### 4.7. Effect of Crude Extracts on Intracellular ROS in m-1 Cells

The method of Wen et al. was employed to determine the intracellular ROS [46]. The ROS was determined using the ROS Reactive Oxygen Species Assay Kit (CA1420, Beijing Solarbio Science and Technology Co., Ltd., Beijing, China).

The m-1 was inoculated in PDB and incubated in a shaker at 37 °C and 120 rpm for 12 h. A total of 18 mL of the bacterial solution was divided in half into a control group and a treatment group. A total of 1 mL of sterile water was added to control and 1 mL of 10 times MIC crude extract was added to treatment, and the two groups were incubated in a shaker at 37 °C and 120 rpm. Samples were taken at 0 h, 2 h, and 4 h to determine the effect of the crude extract on the ROS of m-1. Samples were taken three times in parallel with control and treatment to minimize errors due to differences in the total number of cells in the two sets of samples.

Each sample was centrifuged at 6000 rpm for sediment extraction. The sediment was washed with PBS and resuspended. A total of 200 μL of bacterial solution with the addition of 1 μL of red fluorescent dye solution was mixed well by blowing and incubated at 37 °C for 30 min. The solution was centrifuged at 3000 rpm for 10 min. The supernatant was discarded to remove the dye. The precipitate was washed 2–3 times with PBS to reduce the residue of the dye. The red fluorescence value was measured using the fluorescence photometer by setting the excitation wavelength to 518 nm and the emission wavelength to 610 nm.

### 4.8. Effect of Crude Extract on Intracellular SOD Activity in m-1 Cells

The SOD activity detection kit (BC0170, Beijing Solarbio Science & Technology Co., Ltd., Beijing, China) was used for the determination of SOD. At the same time, the enzyme activity unit of this experiment was stipulated as the following: when the inhibition percentage in the xanthine oxidase coupling reaction system of 50%, the SOD enzyme activity in the reaction system is defined as an enzyme activity unit.

The reaction system was prepared according to the method of Section 4.6.1 and the samples of crude extract were obtained at 0 h, 2 h, and 4 h. The SOD enzyme solution was prepared according to the method of Section 4.6.2. According to the requirements of the test box, the enzyme solution was treated with chemical reagents, the OD_560_ value of the sample was measured with an ultraviolet spectrophotometer, and the corresponding enzyme activity was calculated according to the rules. The test was repeated three times.

### 4.9. Identification of Substances in Crude Extracts

SEMI-Preparative High-Performance Liquid Chromatography (SEMI-HPLC) was used to separate the crude extract to obtain samples. The samples were then identified by QTOF-MS/MS.

The JZ crude extract was filtered through a 0.22 μm filter membrane to remove impurities and used as an analytical sample for SEMI-HPLC. Water and acetonitrile were sonicated for 30 min to remove air bubbles. The column was a Waters Acquity UPLC^®^BEH C18 (100 mm × 2.1 mm × 1.7 μm), mobile phase A was water, and mobile phase B was acetonitrile. Linear gradient elution was from 60–85% and the flow rate was 10 mL/min. The injection volume was 300 μL. Liquid chromatography analysis was performed and the eluent from the first second to the last second of the elution peak was collected as a sample solution for QTOF-MS/MS.

The water in the sample solution was evaporated by the rotary evaporator and then dissolved in methanol to obtain a semi-prepared liquid, which was analyzed by QTOF-MS/MS. The separation was performed using a C18 reversed-phase LC column (2.1 mm × 100 mm). Mobile phase A was water, mobile phase B was acetonitrile, and both mobile phases were supplemented with 0.02% formic acid and 0.05% ammonia. Linear gradient elution was carried out with the following procedure: 0.0–10.0 min, 40–80% B, 10.0–15.0 min, 80–95% B. The injection volume was 4 μL and the flow rate was 0.30 mL/min. The column temperature was 40 °C. MS fragments were analyzed using a gas chromatography system connected to an atmospheric gas chromatography ion source (APGC) and using MassLynx mass spectrometry software (version 4.2.0) in conjunction with the UNIFI Scientific Information System (version 1.9.13.9) to process accurate mass data and structural analysis. The APGC ion source was used in positive ion mode. The instrument parameters were set as follows: capillary voltage (kV): ES+ 3.0 KV, sample cone: 40 V, source setting: 80 V, source temperature: 120 °C, desolvation temperature: 400 °C, cone gas: 50 L/h, desolvation gas: 800 L/h. Collision energy, TOF MS experiment was as follows: low energy 6 V, high energy 20–35 V. Information Dependent Acquisition software (version 1.9.13.9) was used for a single-run analysis with an *m*/*z* range of 100–1500 in TOF MS.

## Figures and Tables

**Table 1:** Kirby–Bauer test to detect the inhibition rate of Bacillus fermentation broth on pathogenic bacteria (%).

Strain	m-4	a-3	m-6	m-1
*Bacillus velezensis*	43.45 ± 2.39	69.9 ± 1.93 **	35.99 ± 5.61 **	47.43 ± 2.22 **
*Bacillus subtilis*	44.63 ± 2.63	59.58 ± 1.32	27.41 ± 4.49	34.24 ± 3.67
*Bacillus amyloliquefaciens*	43.16 ± 2.59	51.11 ± 1.92	25.24 ± 4.16	41.38 ± 4.88

Values are the mean ± standard deviation of the results of at least nine replicates. Asterisks indicate a significant difference (** *p* < 0.01, Duncan’s multiple range test). Strains: m-4, *Brevibacterium bifidum*; a-3, *Bacillus proteolyticus*; m-6, *Pseudomonas bohemia*; m-1, *Bacillus altitudinis.*

**Table 2:** Morphological and biochemical characteristics of strain JZ.

Characteristic	Result	Characteristic	Result
Shape	Short rod	Maltose	+
Cell size (μm)	0.4 × 0.6–1.0 × 3.0	Lactose	+
Mobility	+	Xylose	−
Aerobic	+	Cellulose	−
Gram Staining	+	H_2_S	−
Citrate	+	Methyl red	+
Gelatin liquefaction	+	Amylohydrolysis	+
Calcium phosphate	−	Siderophore	+
Iron (iii) phosphate	−	Lecithin	−
Aluminium phosphate	−	Indole acetic acid	+
Casein	+		

Note: +, positive; −, negative.

**Table 3:** QTOF-MS/MS analysis of the JZ crude extract.

Lipopeptides	Mode	Current Experimental Mass	Previously Reported Mass	References
Surfactin	[M+H]^+^	1022.68, 1036.68, 1050.07/1051.07	1022.67	Toral. et al. (2018) [24]
Iturin	[M+H]^+^	1043.55/1044.55	1043.50	Zhao. et al. (2018) [25]
Fengycin	[M+H]^+^	1449.79, 1463.80, 1477.82, 1491.83	1491.84	Toral. et al. (2018) [24]
Bacillaene	[M+H]^+^	567.03, 581.70	581.50	Xu. et al. (2014) [26]

**Table 4:** Reaction system for the determination of minimum inhibitory concentration (MIC).

Treatment	CK	A	B	C	D	E
Inoculum concentration	1 mL	1 mL	1 mL	1 mL	1 mL	1 mL
PDB	17 mL	17 mL	17 mL	17 mL	17 mL	17 mL
Extract	0 mL 0.0 C	2 mL 0.2 C	2 mL 0.2 C	2 mL 0.1 C	2 mL 0.05 C	4 mL 1.0 C
Sterile water	2 mL	0 mL	0 mL	0 mL	0 mL	0 mL

## Data Availability

The original contributions presented in the study are included in the article, further inquiries can be directed to the corresponding authors.
